# Electromyography Parameter Variations with Electrocardiography Noise

**DOI:** 10.3390/s22165948

**Published:** 2022-08-09

**Authors:** Kang-Ming Chang, Peng-Ta Liu, Ta-Sen Wei

**Affiliations:** 1 Department of Computer Science and Information Engineering, Asia University, Taichung 41354, Taiwan; 2 Department of Digital Media Design, Asia University, Taichung 41354, Taiwan; 3 Department of Medical Research, China Medical University Hospital, China Medical University, Taichung 40402, Taiwan; 4 Graduate Institute of Biomedical Engineering, National Chung Hsing University, Taichung 40227, Taiwan; 5 Fall Prevention Center and Department of Physical Medicine & Rehabilitation, Changhua Christian Hospital, Changhua 500209, Taiwan

**Keywords:** electromyography, electrocardiography, noise

## Abstract

Electromyograms (EMG signals) may be contaminated by electrocardiographic (ECG) signals that cannot be easily separated with traditional filters, because both signals have some overlapping spectral components. Therefore, the first challenge encountered in signal processing is to extract the ECG noise from the EMG signal. In this study, the EMG, mixed with different degrees of noise (ECG), is simulated to investigate the variations of the EMG features. Simulated data were derived from the MIT-BIH Noise Stress Test (NSTD) Database. Two EMG and four ECG data were composed with four EMG/ECG SNR to 32 simulated signals. Following Pan-Tompkins R-peak detection, four ECG removal methods were used to remove ECG with different compensation algorithms to obtain the denoised EMG signal. A total of 13 time-domain and four frequency-domain EMG features were calculated from the denoised EMG. In addition, the similarity of denoised EMG features compared to clean EMG was also evaluated. Our results showed that with the ratio EMG/ECG SNR = 10 and 20, the ECG can be almost ignored, and the similarity of EMG features is close to 1. When EMG/ECG SNR = 1 and 2, there is a large variation of EMG features. The results of our simulation study would be beneficial for understanding the variations of EMG features upon the different EMG/ECG SNR.

## 1. Introduction

The electromyography (EMG) signal represents the muscle performance of central and peripheral motor control, especially EMG collected from back muscles, which can be used to evaluate pain around the shoulder, neck, and back. EMG can also be used as an indicator to evaluate ergonomic performance and trunk performance. Surface EMG (sEMG) is also used for body motion detection, such as hand motion detection [1], hand gesture recognition [2], and shoulder motion intention detection, with the aid of a pattern recognition process [3]. However, the collection of EMG signals is easily disturbed by many noises, which affect the interpretation of EMG results and the accuracy of diagnosis. The noise sources of EMG include motion noise, heartbeat, and respiration. The distance between the recording electrode of EMG and the heart may affect the strength of heartbeat noise on EMG, which increases the complexity of EMG signal processing. Therefore, noise processing techniques, such as high-pass filters, are required for further EMG signal processing. The EMG signal is a time series with constantly changing frequency and amplitude with no specific waveform, therefore many time domains, frequency domains, and nonlinear parameters were developed to analyze the EMG signal characteristics [4,5,6,7]. Different EMG parameters have their prominent effects under different EMG measurement environments. Time-domain characteristic parameters are usually mainly the average value, maximum value, summation, or variation of amplitude. This time domain is inclusive of the average amplitude absolute value (AAV), mean absolute value (MAV1 type-1), average amplitude change (AAC), the maximum value of the amplitude contains the amplitude of the first burst (AFB), the sum of the amplitudes contains integrated EMG (IEMG), simple square integral (SSI), and waveform length (WL). The amplitude variation parameters include standard deviation (STD), root mean square (RMS), log detector (LOG), median differential value (MDV), and difference absolute standard deviation value (DASDV). Zero crossing (ZC) computes the signal period in the time domain. Frequency-domain parameters are usually converted from EMG signal to frequency domain through Fourier Transform, and then its total frequency power (TTP), median frequency (MDF), max peak frequency (PKF), the amplitude of peak frequency (PKF.amp), and so on, are calculated. In addition to Fourier transform, there are other transformation functions, such as Wavelet transform [8], Hilbert-Huang Transform, etc. [9]. In addition to time-domain and frequency-domain parameters, nonlinear parameters also play an important role in EMG’s characteristics [10].

The most common EMG noise is electrocardiography (ECG). There have been many studies trying to separate the ECG signal from the EMG signal, such as the ICA (independent component analysis) separation technique [11]. Abbaspour et al. use the filter combination technique to detect QRS and construct an ECG signal template; drift noise is removed by a low-pass filter, and denoised EMG is obtained by the subtraction of the ECG template. Their method has achieved impressive results [12]. Christov et al. also used a similar filter combination concept to separate ECG and EMG by dynamic filtration [13,14]. The concept of template subtraction is also used in many studies [15,16]. Combining template subtraction and empirical mode decomposition (EMD) is a recent and very innovative approach [17]. Another approach to separate ECG from EMG is to treat ECG as signal and EMG as noise and remove ECG with R-peak detection. Many interesting algorithms have been proposed and the R-peak detection performance is usually examined with public databases, such as MIT-BIH arrhythmia and Fantasia databases. The moving average (MA) filtering method proposed by Modak et al. has achieved 99.82% sensitivity, 99.88% positive predictivity, and 0.31% detection error rates on R-peak detection performance with the MIT-BIH arrhythmia database [18]. The Zhang et al. algorithm achieved a sensitivity of 99.70% and positive predictivity of 99.93% on R-peak detection performance with R-peak threshold [19]. For noise signal processing, MIT-BIH provided a set of noise-stress test databases in 2000 (noise-stress test database, NSTD), with various SNR ECGs with EMG noise, as well as clean EMG and other noise signals [20]. Other methods, such as event-related moving averages [21], and Ensemble Empirical Mode Decomposition achieved great R-peak detection performance [22]. Table 1 organized various ECG-detection algorithms proposed in recent years, applied under NSTD, and their corresponding ECG R-peak detection performance by sensitivity (Se%) and positive predictivity (+P%). From the 1985 Pan-Tompkins algorithm to Rahul et al. in 2021, sensitivity and positive predictivity increased from (74.46%, 93.67%) to (97.58%, 96.04%); The better the R-peak detection performance, the more complex the algorithm is.

Since EMG is an evaluation tool commonly used in many clinical examinations or sports assessment, such as upper back pain and posture adjustment, the EMG electrodes are placed very close to the upper back, for example, EMG is recorded on specific muscles such as the Trapezius muscle. The artifact from the heartbeats is inevitable during activity. It is necessary to extract cardiac artifacts from it and obtain a clean EMG signal. Therefore, the aim of this study was to explore the variations of common time-domain and frequency-domain EMG parameters under different EMG/ECG SNR with conventional Tompkins R-peak detective and removing algorithm.

## 2. Materials and Methods

### 2.1. Simulated EMG and ECG Dataset

**Figure 1:**
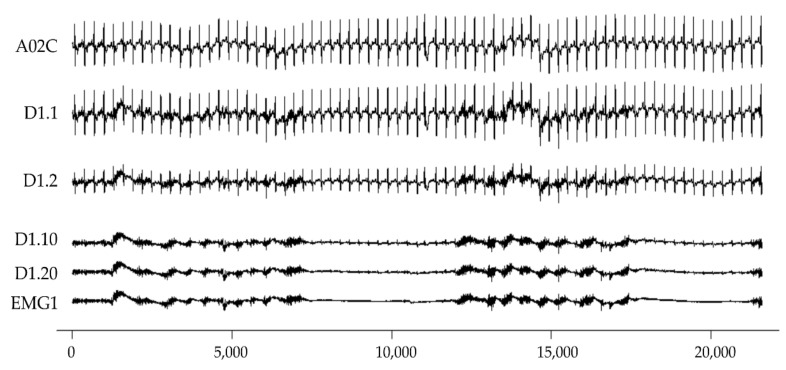
Illustration of simulated EMG and ECG signal. From top to bottom are clean A02C, D1.1, D1.2, D1.10, D1.20, and EMG1. The *x*-axis is sample points; the sampling frequency is 360 Hz. The *y*-axis is an arbitrary unit.

Original data are derived from the MIT-BIH Noise Stress Test (NSTD) Database [30]. The NSTD database consists of 30 min ECG, EMG, and electrode motion artefact recording, respectively. In this study, the first minute of two ECG records (118e00 and 119e00) are used. A0 is derived from 118e00, and B0 is derived from 119e00. Clean ECG is derived from two filters. The first step is to remove the DC component. The second step is to pass through a FIR low pass filter (the order = 36, normalized cutoff frequency = 0.1, hamming window). The filter coefficients are implemented by fir1 function in a signal package with R languages. The two-channel ECG signals of A0 form A02C and A03C; the same process of B02C and B03C is derived from B0. EMG is taken from the ma signals in the NSTD database, which are EMG1, and EMG2; the mixed signals are combined according to the four EMG/ECG intensity ratios: 1, 2/1, 10/1 and 20/1. The abbreviations are Dm.1, Dm.2, Dm.10 and Dm.20, with corresponding SNR (signal-to-noise ratio) are 0 dB (1:1), 6 dB (2:1), 20 dB (10:1) and 26 dB (20:1). SNR is calculated using Equation (1). Table 2 is the list of the mixed signals. Figure 1 is an illustration of some mixed EMG signals. The original signal file and subsequent EMG feature estimation code are provided in Appendix A.

(1) $$\mathrm{SNR}=20{\;*\;\log}_{10}\left(\frac{\mathrm{EMG}}{\mathrm{ECG}}\right)$$

### 2.2. ECG R-Peak Detection

ECG R-peak detection is based on the Pan-Tompkins algorithm [31], and implemented by the R rsleep package, with detect rpeaks code to estimate R-peak location [32], and then calculates the median and standard derivation of R-peak time series. The Pan-Tompkins method consists of a band-pass filter, following with derivatives, squaring, and moving window integration. The R-peak position is determined with a threshold. For the clean ECG dataset, A02C, A03C, B02C, and B03C, the numbers of detected R peaks are used as the reference to compare the calculated R peaks of the corresponding mixed EMG signals. Due to the different SNR, some R peaks will be missing, or some EMG bursts will be misjudged as R peaks, so the following indices are used to evaluate the R-peak detection ability.

True positive (TP): the number of QRS correctly identified.(2)

False-positive (FP): peaks that do not correspond to the QRS.(3)

False-negative (FN): the number of QRS incorrectly identified.(4)

Sensitive percentage (Se%) = TP/(TP + FN)(5)

Positive Predictivity rate (+P%) = TP/(TP + FP)(6)

**Table 2:** Simulated signals with EMG mixed ECG components, m represented as series of signal, which is 1 to 8.

EMG	m	ECG	Dm.1	Dm.2	Dm.10	Dm.20
			EMG + ECG × 1	EMG + ECG × 0.5	EMG + ECG × 0.1	EMG + ECG × 0.05
EMG1	1	A02C	D1.1	D1.2	D1.10	D1.20
	2	A03C	D2.1	D2.2	D2.10	D2.20
	3	B02C	D3.1	D3.2	D3.10	D3.20
	4	B03C	D4.1	D4.2	D4.10	D4.20
EMG2	5	A02C	D5.1	D5.2	D5.10	D5.20
	6	A03C	D6.1	D6.2	D6.10	D6.20
	7	B02C	D7.1	D7.2	D7.10	D7.20
	8	B03C	D8.1	D8.2	D8.10	D8.20

### 2.3. ECG Deletion

**Figure 2:**
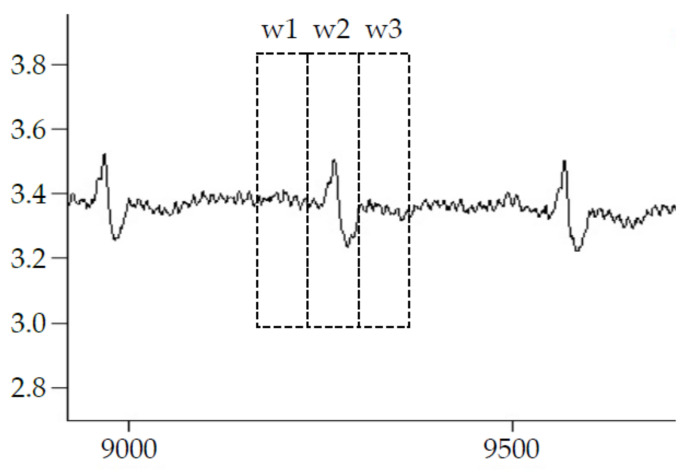
Illustration of ECG deletion method.

After detecting the R-peak locations in the EMG/ECG simulated signal, the following four methods are used to remove the components of the ECG signal to obtain a denoised EMG signal. Figure 2 depicts the four ECG deletion methods implemented in this study.

F0 Method: No signal processing at all.

F1 method: The detected R peak is centered on the window w2, which is covered by a 0.2 sec window width. The signal within window 2 is set as 0.

F2 method: The detected R peak is centered on the window w2, then the signal within the w2 is replaced as the average of w1 and w3. The duration of w1, w2, and w3 are 0.2 s.

F3 method: After EMD decomposes the mixed EMG signals, it takes the first two layers of IMF (intrinsic mode function), IMF1, and IMF2 signals, and recombines them. It is like a high-pass filtering approach to remove ECG contents.

### 2.4. EMG Parameters

There are thirteen time-domain and four frequency-domain EMG features investigated in the study. The list of EMG features and the corresponding formula are as shown in Table 3. In order to understand the degree of EMG parameter variation, the similarity index (SI) is defined as below:

SI = EMG feature derived from simulated mixed EMG/EMG feature derived from raw clean EMG.

### 2.5. Statistics

**Figure 3:**
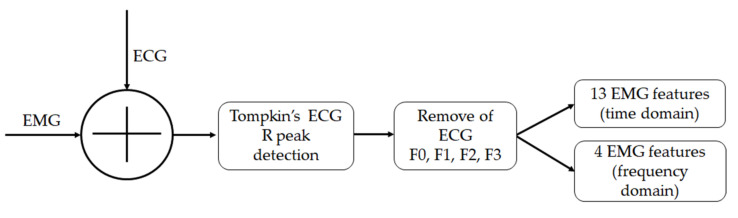
Experiment flowchart.

Mean and standard deviation of the following time series were calculated: the numbers of R peaks were detected in the simulated signal, time-domain and frequency-domain of EMG features, and the ratio of its value corresponding to the clean EMG. The paired *t*-test was used to examine the difference of the EMG features among the four ECG deletion processing methods (F0–F1, F0–F2, F0–F3, F1–F2, F1–F3, and F2–F3). The *p*-value was set to 0.05. The experiment flowchart of this study is shown in Figure 3.

**Table 3:** EMG features and corresponding formula, where *x*(*t*) is denoised EMG signal.

**Number**	**Feature Name (Abbreviation)**	**Formula**
**Time-Domain**		
7	Average amplitude abs value (AAV)	$\frac{1}{N}\sum_{t=1}^{N}\left|X_{t}\right|$
8	Standard deviation (STD)	$\sqrt{\frac{1}{N\;-\;1}\sum_{t=1}^{N}X_{t}{}^{2}}$
9	Integrated EMG (IEMG)	$\sum_{t=1}^{N}\left|X_{t}\right|$
10	MAV1 type-1	$\frac{1}{N}\sum_{t=1}^{N}W_{n}\left|X_{t}\right|$ $W_{n}=\left\{\begin{array}{c} 1,\;if\;0.25N\leq n\leq 0.75N\\ 0.5,\;\;\;otherwise \end{array}\right.$
11	Simple square integral (SSI)	$\sum_{t=1}^{N}{\left|X_{t}\right|}^{2}$
12	Root mean square (RMS)	$\sqrt{\frac{1}{N}\sum_{t=1}^{N}X_{t}{}^{2}}$
13	LOG (log detector)	$\frac{1}{N}\sum_{t=1}^{N}log\left(\left|X_{t}\right|\right)$
14	Waveform length (WL)	$\sum_{t=1}^{N-1}\left|X_{t+1}-X_{t}\right|$
15	Average amplitude change (AAC)	$\frac{1}{N}\sum_{t=1}^{N-1}\left|X_{t+1}\;-\;X_{t}\right|$
16	Median differential value (MDV)	$\frac{1}{N}\sum_{t=1}^{N-1}\left[X_{t+1}-X_{t}\right]$
17	Difference absolute standard deviation value (DASDV)	$\sqrt{\frac{1}{N\;-\;1}\sum_{t=1}^{N\;-\;1}{\left(X_{t+1}-X_{t}\right)}^{2}}$
18	Amplitude of the first burst (AFB)	$max\left(X_{t}\right)$
19	Zero crossing (ZC)	$\sum_{t=1}^{N\;-\;1}\left[sgn\left(X_{t}\;\times \;X_{t+1}\right)\;\cap \;\left|X_{t}-X_{t+1}\right|\geq thershold\right]$ $sgn\left(X\right)=\left\{\begin{array}{c} \;1,\;if\;X\geq \;threshold\\ 0,\;\;\;\;\;\;\;\;\;\;\;\;\;\;otherwise \end{array}\right.$
**Number**	**Feature Name (Abbreviation)**	**Formula**
**Frequency-Domain**		
20	Total power (TTP)	$\sum_{t=1}^{M}P_{t}$
21	median frequency (MDF)	$\sum_{t=1}^{MDF}P_{t}=\sum_{t=MDF}^{M}\;P_{t}=\;\frac{1}{2}\sum_{t=1}^{M}P_{t}$
22	Max peak frequency (PKF)	$\max\;(P_{t}$), *t* = 1, …, *M*
23	Amplitude of peak frequency (PKF.amp)	$\mathrm{amp}\;\mathrm{at}\;\max\;(P_{t}$), *t* = 1, …, *M*

## 3. Results

### 3.1. R-Peaks Detection Performance

The R-peaks detection performance of four simulated signal is listed in Table 4. For ECG A02C, the true R-peak number is 73. The numbers of R peaks detected from the simulated EMG: D1.1.R, and D1.2.R are same as 73, but D1.10.R and D1.20.R drop to 55 and 32, respectively. The accurate median RR interval is 0.836 sec, and the corresponding median RR interval for D1.1.R to D1.20.R is 0.830, 0.830, 0.433, and 0.294. The above results show that ECG signal ratios are smaller than EMG under conditions of D1.10.R and D1.20.R, so it is not easy to detect true R peak, even by increasing FP and FN. The other three ECGs (A03C, B02C and B03C) have similar results.

### 3.2. Similarity for All Features in Different EMG/ECG SNR

Regardless of the ECG deletion methods, the time-domain and frequency-domain EMG features on 16 simulated EMG signals are averaged. The selected features are listed in Table 5. Table 5 shows that, compared with the true EMG (EMG1), the order of the time-domain features are Dm.1 > Dm.2 > Dm.10 > Dm.20 ≥ true EMG.

To compare the similarity of EMG features between simulated EMG and true EMG, the ratio of EMG feature to the true EMG is defined as a similarity index (SI). If this ratio is close to 1, it means the EMG feature of the simulated mixed signal is exactly the same as that of the raw true EMG. The mean and standard deviation of the ratios of all time-domain parameters are calculated and listed in Table 6. With a single-sample *t*-test to test whether there is a difference with ratio = 1, there is no statistical difference with ratio = 1 for F2 at Dm.10, and the mean and standard deviation of the ratio are 0.997 and 0.035, respectively. This means that by using F2 ECG deletion method under SNR = 20dB (Dm.10) condition, the EMG features have the highest similarity with features calculated from true EMG.

To understand the influence of the four ECG deletion methods on the EMG features, the paired *t*-test was used to test the SI difference among F0, F1, F2, and F3 on Dm.1 to Dm.20. The results are shown in Table 7. The findings reveal that F0 has significant differences from F1, F2, and F3 in Dm.1, Dm.2, Dm.10, and Dm.20, and the SI of F0 is also higher than that of F1, F2 and F3, which means that there is the highest derivation on F0 than the other three methods, since there is no removal of ECG components. The F1 method is to delete the signal value within the window length centered at the R peak. F2 is to replace the signal within the window with average EMG around the window. There are significant differences among F0, F1, and F2. F3 is a high-pass filter, and the result of F3 is close to F1. There is no statistical difference between F1 and F3 in Dm.1, Dm.10, and Dm.20. From Table 6, the SI from Dm.1 to Dm.2 are 1.986 (4.927) to 1.299 (1.164) for F1, but it is 1.287 (0.652) to 0.669 (0.373) for F3. The SI for F3 is almost always <1, after Dm.1. Therefore, the disadvantage of the F3 ECG deletion method is that it will greatly suppress the original EMG signal parameters’ performance. In Table 6, the first row of ALL signal is used to demonstrate the SI from Dm.1 to Dm.20. Paired *t*-tests among Dm.1 to Dm.20 of ALL signals are all statistical differences. In other words, different EMG/ECG ratios do affect the degree of variation in EMG features.

### 3.3. Similarity for Time-Domain Features in Different EMG/ECG SNR

Table 8 contains the details of the SI distribution on each time-domain feature for F0, F1, F2, and F3. Combined with Table 6, the variations of SI on each EMG feature among F0 to F3 with different SNR were clearly shown. As shown in Table 8A–C, there was a trend of Dm.1 >Dm.2 >Dm.10 >Dm.20 for all time-domain EMG features. Most SI of time-domain features are below 2; that of AAV, STD, IEMG, MAV1, RMS, and LOG are around 2~3. The two highest features are SSI (SI = 12.8) and AFB (SI = 22.4). In other words, these two time-domain EMG features are particularly affected by ECG noise interference. In addition, the F3 method will filter out some EMG components, the SI are <1, as shown in Table 8D.

### 3.4. Similarity for Frequency-Domain Features in Different EMG/ECG

**Figure 4:**
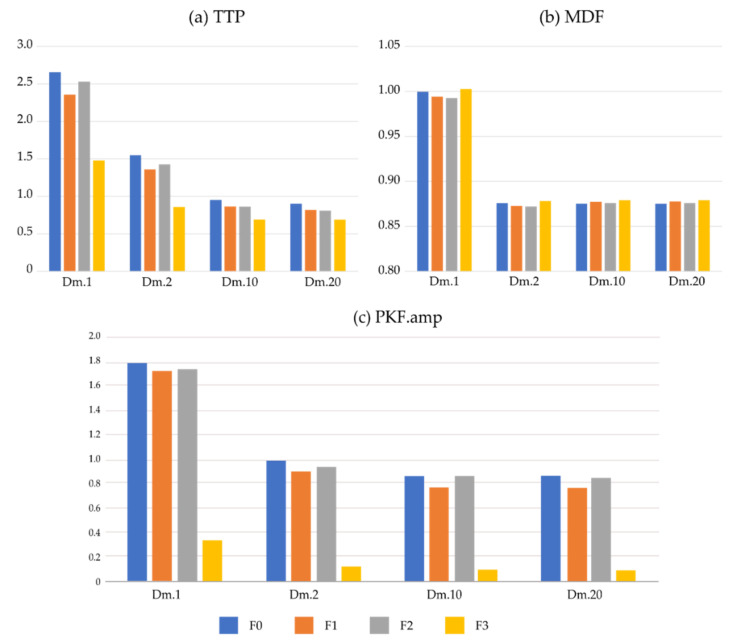
The similarity index of frequency-domain features. TTP: total power, MDF: median frequency, PKF. amp: amplitude of peak frequency.

Table 9 lists the average PKF value among four SNR conditions. PKF is interfered with by ECG noise, and the PKF ratio even exceeds 100. Therefore, PKF is a feature with great variation under noise. Figure 4 directly lists ratios for three frequency-domain features. Unlike the results of time-domain features, there is no consistent rule for frequency-domain features on the SI which vary greatly on each frequency-domain feature. The performance of TTP is similar to traditional time-domain parameters. For MDF, the SI is close to 0.87, and is similar on Dm.2, Dm.10, and Dm.20, regardless of the four ECG removing methods. The SI is almost = 1 for Dm.1, which is the simulated signal with the highest ECG noise. This shows that MDF is very robust for ECG noise.

## 4. Discussion

This study discusses the change in EMG parameters in the presence of mixed heartbeat noise. There were several studies on heartbeat signal removal from EMG, such as the subtraction method [33], adaptive filters [34], independent component analysis (ICA), singular spectrum analysis and segmented-beat modulation method [35]. For example, Barrios used the singular spectrum analysis (SSA) to remove ECG without using the ECG reference signal and it outperformed the other methods [36]. Thandar used wavelet transform to compare 9 SNR of EMG/ECG (−20 to 20 dB, in increments of 5 dB) [37], and removed ECG noise by discrete wavelet transform. Thandar’s research is similar to this study. Their results found that discrete wavelet transform is better than high-pass filtering in terms of relative error, correlation coefficient, and absolute mean error. Different thresholds are required to remove ECG noise with different SNRs. It is not practical to apply to real-time systems since it takes too much time to find out optimal thresholds. Therefore, they suggest that future research can develop a method for estimating SNR to speed up the noise-removing signal processing procedure. Abbaspour et al. combined wavelet and artificial neural networks to remove ECG from EMG signals [38]. According to the above studies, if the SNR of EMG is known in advance, it can enhance the estimation algorithm performance. Although this study only compares four common ECG removal methods, with the traditional Pan-Tompkins R-peak detection algorithm. The major contribution is to understand the derivation degrees of the extracted EMG parameters. Subsequent research can develop a system for predicting ECG noise, followed by an optimal ECG filtering algorithm, and it is believed that better EMG estimation results can be obtained. In this study, Pan-Tompkins algorithm was used to detect the R peak. According to the study [39], the R-peak performance (Se%, +P%) with the Pan-Tompkins algorithm on the NSTD Database, with the noisiest two sets of signals of 118e_6 and 119e_6, were (71.47%, 60.50%) and (66.48%, 54.21%), respectively. It is very similar to the Dm.10 results demonstrated in Table 4. This means that the R-peak detection result in this study is close to [39] and is acceptable. The main contribution of this paper is to discuss the EMG parameters’ changes under EMG/ECG SNR. For different clinical EMG parameters, the results of this paper can be used to estimate whether clinical EMG features are derived from real EMG or from ECG noise. In addition, although it can be used to estimate EMG/ECG SNR, it is not the focus of this article, but this article gives a good direction for a follow-up study to estimate the EMG/ECG SNR in time, with a further rigorous examination.

## 5. Conclusions

This study uses simulated EMG and ECG to demonstrate the changes in the time-domain or frequency-domain EMG features under the different EMG/ECG SNR. When the ECG signal strength is relatively lower than EMG, it is only necessary to (1) detect and delete the ECG, and then (2) compensate with the average value of the left and right windows of EMGs, and then (3) the EMG features close to the original EMG can be obtained; different EMG features have different degrees of change after noise interference. Since different EMG features are suitable for different EMG measurement environments, this study can provide a reference for various signal processing of EMG users.

## Figures and Tables

**Table 1:** Average R-peak detection performance on MIT-BIH noise stress test database (NSTD).

Author	Year	Se%	+P%
Pan-Tompkins [12]	1985	74.46	93.67
Benitez DS, et al. [23]	2000	93.48	90.60
Li, H. and Tan, [24]	2006	90.66	87.19
Plesnik et al. [25]	2012	72.11	82.48
Elgendi, M [26]	2013	95.39	90.25
Dohare, et al. [27]	2014	88.20	89.19
Yakut, Ö. and Bolat, E. D. [28]	2018	93.62	94.52
Rahul, J., et al. [29]	2021	97.58	96.04

**Table 4:** R-peak detection performance of simulated ECG. (A) A02C, (B) A03C, (C) B02C, (D) B03C.

**A**							
**ECG**	**TP**	**FP**	**FN**	**Se%**	**+P%**	**Median (Sec)**	**STD (Sec)**
A02C	73	n.a.	n.a.	100	100	0.836	0.042
D1.1.R	73	0	0	100	100	0.830	0.045
D1.2.R	73	1	0	100	98.6	0.830	0.087
D1.10.R	55	35	18	75.3	61.1	0.433	0.791
D1.20.R	32	30	41	43.0	51.6	0.294	2.183
D5.1.R	73	0	0	100	100	0.833	0.048
D5.2.R	73	3	0	100	96.1	0.833	0.133
D5.10.R	30	22	43	41.1	57.7	0.286	2.021
D5.20.R	24	33	49	32.9	42.1	0.254	2.028
**B**							
**ECG**	**TP**	**FP**	**FN**	**Se%**	**+P%**	**Median (Sec)**	**STD (Sec)**
A03C	73	n.a.	n.a.	100	100	0.836	0.032
D2.1.R	73	0	0	100	100	0.833	0.031
D2.2.R	72	4	1	98.6	94.7	0.825	0.132
D2.10.R	33	34	40	45.2	49.3	0.313	2.091
D2.20.R	30	25	43	41.1	54.5	0.295	2.128
D6.1.R	73	0	0	100	100	0.836	0.034
D6.2.R	72	16	1	98.6	81.8	0.811	0.233
D6.10.R	40	19	33	54.8	67.8	0.255	1.989
D6.20.R	25	36	48	34.2	41	0.243	1.929
**C**							
**ECG**	**TP**	**FP**	**FN**	**Se%**	**+P%**	**Median (Sec)**	**STD (Sec)**
B02C	65	n.a.	n.a.	100	100	0.897	0.296
D3.1.R	65	1	0	100	98.5	0.9	0.298
D3.2.R	65	1	0	100	98.5	0.897	0.298
D3.10.R	39	35	26	60	52.7	0.477	0.733
D3.20.R	25	39	40	38.5	39.1	0.291	2.143
D7.1.R	65	0	0	100	100	0.897	0.296
D7.2.R	65	4	0	100	94.2	0.881	0.311
D7.10.R	29	37	36	44.6	43.9	0.377	0.926
D7.20.R	19	39	46	29.2	32.8	0.261	1.971
**D**							
**ECG**	**TP**	**FP**	**FN**	**Se%**	**+P%**	**Median (Sec)**	**STD (Sec)**
B03C	66	n.a.	n.a.	100	100	0.897	0.303
D4.1.R	65	1	1	98.5	98.5	0.894	0.305
D4.2.R	61	3	5	92.4	95.3	0.883	0.414
D4.10.R	27	43	39	40.9	38.6	0.305	1.337
D4.20.R	23	42	43	34.8	35.4	0.275	2.141
D8.1.R	63	3	3	95.5	95.5	0.886	0.372
D8.2.R	50	17	16	75.8	74.6	0.863	0.619
D8.10.R	20	39	46	30.3	33.9	0.258	1.961
D8.20.R	19	43	47	28.8	30.6	0.254	1.927

**Table 5:** Selected EMG of features in time-domain.

Features	D1.1	D1.2	D1.10	D1.20	EMG1
AAV	0.2668	0.1555	0.0859	0.0813	0.0798
STD	0.3152	0.1665	0.0817	0.0792	0.0787
IEMG	5789.02	3362.19	1862.9	1766.47	1730.78
MAV	0.268	0.1556	0.0862	0.0817	0.0801
MAV1	0.2704	0.1545	0.0821	0.0773	0.0755
SSI	3685	1121.82	303.84	278.63	270.53
VAR	0.1666	0.0124	0.0008	0.0008	0.0008
ZC	52	54	40	32	30

**Table 6:** The similarity index (SI) of the time-domain of EMG features in processed EMG signals. Data are represented as mean (standard deviation). m = 1 to 8.

Signals	Dm.1	Dm.2	Dm.10	Dm.20
ALL	1.760 (4.569)	1.300 (1.145)	0.978 (0.247)	0.944 (0.236)
F0	2.877 (6.122)	1.763 (1.371)	1.069 (0.043)	1.015 (0.017)
F1	1.986 (4.927)	1.299 (1.164)	0.874 (0.107)	0.852 (0.096)
F2	2.156 (4.878)	1.438 (1.124)	0.997 (0.035)	0.953 (0.035)
F3	1.287 (0.652)	0.669 (0.373)	0.529 (0.402)	0.528 (0.401)

**Table 7:** Paired *t*-test results among four ECG deletion methods with four different SNR conditions.

Method Pair	Dm.1	Dm.2	Dm.10	Dm.20
F0–F1	0.015	0.001	<0.001	<0.001
F0–F2	0.025	0.006	<0.001	<0.001
F0–F3	0.026	0.006	0.004	0.006
F1–F2	<0.001	<0.001	0.001	0.001
F1–F3	0.059	0.025	0.073	0.111
F2–F3	0.044	0.012	0.012	0.021

**Table 8:** The similarity index of time-domain features of four EMG/ECG SNR, compared to four ECG removing methods. From top to bottom are F0, F1, F2, and F3.

**(A)**				
**F0**	**Dm.1**	**Dm.2**	**Dm.10**	**Dm.20**
AAV	2.881	1.767	1.07	1.022
STD	3.939	2.103	1.044	1.008
IEMG	2.877	1.763	1.069	1.021
MAV1	2.942	1.792	1.073	1.023
SSI	12.872	3.971	1.12	1.03
RMS	3.411	1.93	1.058	1.015
LOG	2.48	1.629	1.082	1.031
WL	1.529	1.218	1.023	1.008
AAC	1.529	1.218	1.023	1.008
MDV	1.44	1.197	0.997	0.994
DASDV	1.466	1.138	1.006	1.001
AFB	22.446	5.943	1.072	1.005
ZC	1.542	1.452	1.148	1.06
**(B)**				
**F1**	**Dm.1**	**Dm.2**	**Dm.10**	**Dm.20**
AAV	1.986	1.299	0.888	0.861
STD	2.628	1.558	0.971	0.94
IEMG	2.001	1.295	0.884	0.857
MAV1	2.036	1.302	0.867	0.836
SSI	5.908	2.138	0.859	0.806
RMS	2.305	1.423	0.926	0.898
LOG	1.725	1.111	0.685	0.692
WL	1.179	0.985	0.796	0.778
AAC	1.179	0.985	0.796	0.778
MDV	1.033	0.871	0.675	0.636
DASDV	1.753	1.204	0.874	0.852
AFB	19.306	5.332	0.952	0.883
ZC	1.56	1.389	1.055	0.992
**(C)**				
**F2**	**Dm.1**	**Dm.2**	**Dm.10**	**Dm.20**
AAV	2.156	1.438	1.017	0.98
STD	2.682	1.572	0.977	0.948
IEMG	2.228	1.456	1.016	0.98
MAV1	2.276	1.475	1.014	0.975
SSI	6.512	2.381	0.997	0.932
RMS	2.418	1.502	0.998	0.965
LOG	1.956	1.387	1.04	0.998
WL	1.362	1.12	0.947	0.925
AAC	1.362	1.12	0.947	0.925
MDV	1.282	1.06	0.989	0.989
DASDV	2.033	1.326	0.936	0.911
AFB	19.239	5.315	0.948	0.88
ZC	1.593	1.397	1.033	0.953
**(D)**				
**F3**	**Dm.1**	**Dm.2**	**Dm.10**	**Dm.20**
AAV	0.944	0.565	0.428	0.426
STD	1.566	0.776	0.631	0.63
IEMG	0.939	0.563	0.427	0.425
MAV1	0.977	0.592	0.45	0.448
SSI	1.717	0.451	0.281	0.28
RMS	1.269	0.669	0.529	0.528
LOG	0.519	0.39	0.301	0.299
WL	1.316	1.086	0.995	0.993
AAC	1.316	1.086	0.995	0.993
MDV	1.287	1.082	0.925	0.923
DASDV	1.219	1.024	0.98	0.98
AFB	3.242	0.59	0.415	0.399
ZC	1.769	1.733	1.687	1.674

**Table 9:** The max. of peak frequency of 4 EMG/ECG SNR, compared to 4 ECG removing methods.

PKF	Dm.1	Dm.2	Dm.10	Dm.20
F0	999.8	541.7	540.7	0.9
F1	5.5	2.4	0.9	0.9
F2	541.8	2.5	540.7	0.9
F3	604.7	693.6	1117.7	667.7

## Data Availability

All the original data are derived from public databases: MIT-BIH Noise Stress Test (NSTD) Database.

## Appendix A

Original signal file and subsequent EMG feature estimation code. https://doi.org/10.6084/m9.figshare.20180327.
